# The Effect of Exogenous Bile Acids on Antioxidant Status and Gut Microbiota in Heat-Stressed Broiler Chickens

**DOI:** 10.3389/fnut.2021.747136

**Published:** 2021-11-24

**Authors:** Chang Yin, Bing Xia, Shanlong Tang, Aizhi Cao, Lei Liu, Ruqing Zhong, Liang Chen, Hongfu Zhang

**Affiliations:** ^1^The State Key Laboratory of Animal Nutrition, Institute of Animal Sciences, Chinese Academy of Agricultural Sciences (CAAS), Beijing, China; ^2^College of Animal Science and Technology, Northwest A&F University, Xianyang, China; ^3^Shandong Longchang Animal Health Care Co., Ltd., Jinan, China

**Keywords:** heat stress, bile acid, antioxidant status, cecal microbiota, broiler chicken

## Abstract

Bile acids are critical for lipid absorption, however, their new roles in maintaining or regulating systemic metabolism are irreplaceable. The negative impacts of heat stress (HS) on growth performance, lipid metabolism, and antioxidant status have been reported, but it remains unknown whether the bile acids (BA) composition of broiler chickens can be affected by HS. Therefore, this study aimed to investigate the modulating effects of the environment (HS) and whether dietary BA supplementation can benefit heat-stressed broiler chickens. A total of 216 Arbor Acres broilers were selected with a bodyweight approach average and treated with thermal neutral (TN), HS (32°C), or HS-BA (200 mg/kg BA supplementation) from 21 to 42 days. The results showed that an increase in average daily gain (*P* < 0.05) while GSH-Px activities (*P* < 0.05) in both serum and liver were restored to the normal range were observed in the HS-BA group. HS caused a drop in the primary BA (*P* = 0.084, 38.46%) and Tauro-conjugated BA (33.49%) in the ileum, meanwhile, the secondary BA in the liver and cecum were lower by 36.88 and 39.45% respectively. Notably, results were consistent that SBA levels were significantly increased in the serum (3-fold, *P* = 0.0003) and the ileum (24.89-fold, *P* < 0.0001). Among them, TUDCA levels (*P* < 0.01) were included. Besides, BA supplementation indeed increased significantly TUDCA (*P* = 0.0154) and THDCA (*P* = 0.0003) levels in the liver, while ileal TDCA (*P* = 0.0307), TLCA (*P* = 0.0453), HDCA (*P* = 0.0018), and THDCA (*P* = 0.0002) levels were also increased. Intestinal morphology of ileum was observed by hematoxylin and eosin (H&E) staining, birds fed with BA supplementation reduced (*P* = 0.0431) crypt depth, and the ratio of villous height to crypt depth trended higher (*P* = 0.0539) under the heat exposure. Quantitative RT-PCR showed that dietary supplementation with BA resulted in upregulation of *FXR* (*P* = 0.0369), *ASBT* (*P* = 0.0154), and *Keap-1* (*P* = 0.0104) while downregulation of *iNOS* (*P* = 0.0399) expression in ileum. Moreover, 16S rRNA gene sequencing analysis and relevance networks revealed that HS-derived changes in gut microbiota and BA metabolites of broilers may affect their resistance to HS. Thus, BA supplementation can benefit broiler chickens during high ambient temperatures, serving as a new nutritional strategy against heat stress.

## Introduction

According to the Food and Agriculture Organization (FAO), chicken meat accounted for 36.5% of all meat produced worldwide (equivalent 117.2 million metric tons), owing to its rich nutrition, low cost, high-quality protein, low cholesterol, and low fat (1). However, with the rapid development and growth of meat-type chickens, particularly sensitive to ambient temperatures, and the fact that heat stress (HS) cannot be ignored (2, 3). The negative impacts of HS on growth performance, lipid metabolism, and redox balance have been reported (4, 5). Our previous study suggested that chronic heat stress caused a reduction in Tauro-conjugated bile acids (TCBA) synthesis, conjugation, and uptake transport in pigs, which was independent of reduced feed intake (6). Bile acids (BA) play important roles in lipid and fat-soluble substances digestion, antioxidant status, and lipid metabolism, etc. (7). The primary BA is synthesized from cholesterol in the liver, then secreted into the intestine lumen primarily as the amino acid conjugates. Around 95% of BA is reabsorbed in the terminal ileum by the ileal bile acid transporter (ASBT, the apical ileal sodium-dependent bile acid cotransporter) and recycled to the liver *via* the enterohepatic circulation (8). The remaining BA is further metabolized to the secondary BA through deconjugation and multistep dehydroxylation reactions carried out by gut bacteria (9, 10). Our previous study suggested that bile acids explained 37.1% of variations in the luminal microbial composition in growing pigs (11). Gut microbiota can deconjugate the glycine and taurine from conjugated bile acids to yield deconjugated bile acids, which is a critical step in microbial bile acid metabolism. Our and other previous studies have reported that the dysbiosis of the gut microbiota induced by environmental stress (HS, NH3, etc.) was associated with the modification of BA structure (12, 13). Therefore, changes in intestinal flora lead to alterations of the BA profiles, in turn, the BA themselves affect the composition of the intestinal flora and modulate host physiology was also indicated (14).

Previous works have focused mainly on dietary high-energy density or applying additives such as electrolytes, minerals, vitamins, and Chinese herbal to exert anti-stress (15). However, “targeted” strategies for alleviating HS get more and more attention. As a new nutritional strategy, dietary supplementation with BA could elevate the activities of lipoprotein lipase and duodenum lipase was previously reported (7). Secondly, the ligand-activated transcription factor farnesoid x receptor (FXR) can also be activated by specific BA metabolites, including cholic acid (CA), chenodeoxycholic acid (CDCA), deoxycholic acid (DCA), lithocholic acid (LCA), or their conjugated metabolites, thus acting as the cellular sensor, it also serves as the crucial metabolic regulator and liver protector (16, 17). Growing evidence suggests that BA are intensively related to the gut microbiota, and they interact with each other (18). For instance, Kang et al. found that the secondary BA (endogenous bile acids) enhanced the inhibitory activity of tryptophan-derived antibiotics, 1-acetyl-β-carboline, and turbomycin A, which appear to inhibit cell division of *Clostridium difficile* (19). There remains a lack of studies investigating whether HS alters the composition of the BA pool and whether exogenous BA can alleviate HS by its characteristics described above. In this study, we considered the cost-effectiveness and in practical applications, a dose of 200 mg/kg BA compound (the components are shown below) was selected as the recommended dose in alleviating HS attracts our attention. Thus, the potential effects of BA on alleviating heat stress in broiler chickens need to be further studied.

## Materials and Methods

### Animals and Experimental Design

The animal component of this study was conducted in accordance with the Guidelines for Experimental Animals, established by the Ministry of Science and Technology (Beijing, China). Experimental procedures related to involving live roosters were approved by the Experimental Animal Welfare and Ethical Committee of the Institute of Animal Sciences, Chinese Academy of Agricultural Sciences (IAS 2019-78, Beijing, China).

For statistical analyses, the treatments were divided into three groups for all bioassays: (1) thermal neutral group (TN), (2) heat stress group (HS), and (3) HS-BA, 200 mg/kg of feed.

The porcine origin of BA compound, which was provided by Shandong Longchang Animal Health Care Co., Ltd., Jinan, China. The composition of the BA compound was analyzed by LC-MS/MS as described (6), which contains THCA (34.02%), UDCA (25.58%), CDCA (16.09%), HDCA (11.23%), GUDCA (9.6%). Day-old Arbor Acres male chicks were reared in cages for 16 days, then all birds were weighed, and 216 broilers near to average weight were selected and divided into three treatments (Supplementary Table S1), birds in each treatment group were randomly allocated into six-floor pens (12 birds each pen). All birds were caged in the environmental control cabins of the State Key Laboratory of Animal Nutrition and had *ad libitum* access to water and feed. After the 5-day adaptation in the environmental control cabins, the temperature of HS and HS-BA groups was increased to 32°C in 2 h on d21, the remaining cabins were maintained at 23°C, and the humidity in all cabins was maintained at 60 ± 5% until d42. Birds in the TN and HS groups were provided a basal diet, while those in the HS-BA group were fed a BA diet including the supplementation mentioned above. Environmental conditions except temperature were referred to the AA broiler management guide and kept the same among all cabins. The experimental diets (Supplementary Table S2) were formulated to meet or exceed the nutritional requirements for broiler chickens calculated according to The National Research Council (NRC, 1994) recommended. On d42, blood samples were withdrawn from the wing of birds (two birds each pen) to separate serum, then stored at −80°C. Before tissue samples collection, all birds were refed for 2 h, two chickens from each pen were sacrificed by over-dose anesthetic injection. Samples were then collected from the liver, ileum, and the contents of ileum and cecum, aliquoted and stored at −80°C until use.

### Apparent Digestibility of Crude Fat

After drying, the crude fat of diets and feces were determined *via* the Soxhlet extractor method (Hanon SOX-406, Jinan, China). The contents of acid-insoluble ash (AIA) in both diets and feces were directly measured according to China National Standard Method (GB/T23743-2009). The apparent digestibility of crude fat was calculated using the following formula: Digestibility of CF (%) = [1 - the CF content in feces/the CF content in diets × (the AIA content in diets × the AIA content in feces)] × 100.

### Oxidative Damages and Antioxidant Activity in Serum and Liver

The activities of malondialdehyde (MDA), superoxide dismutase (SOD), and glutathione peroxidase (GSH-Px) in the serum and liver were measured with commercial kits (Nanjing Jian Cheng Bioengineering Institute, Nanjing, China) following the instructions of the manufacturer. To quantify MDA, SOD, and GSH-Px in the liver, ~100 mg of liver tissue was homogenized in 900 ml of physiological saline to make a 10 w/v% homogenate, and the supernatant was harvested after centrifugation at 2,000 rpm for 10 min at 4°C.

### H&E Staining for Histomorphological Analysis

The ileum samples were dehydrated, embedded in paraffin, and then sliced at 5 μm thickness. The samples were stained with hematoxylin and eosin (HE) and observed on a Leica DM2000 light microscope (Leica Microsystems, Wetzlar, Germany). The images were analyzed with Image J version 1.8 software (National Institutes of Health, MD, USA). Six replicates of complete villus and crypt from each histological section were selected for measurement, and at least four vision fields were chosen.

### LC-MS/MS to Quantify BA in Serum and Liver as Well as Intestinal Contents

The bile acids in serum, liver and intestinal contents were profiled with a Waters Xevo TQ-S LC/MS mass spectrometer (Waters, Milford, MA, USA) equipped with an ESI source and the assay condition used in the previous report by Fang et al. (20). All solvents were of liquid chromatography-mass spectrometry grade. The BA extraction in serum was described by Fang et al. (20), the volume of 200-μl serum was mixed with an equal amount of pre-cold sodium acetate buffer (50 mM, pH 5.6) and triple ethanol. The mixture was then vortexed for 2 min and allowed to stand for 30 min at 4°C, following centrifuged at 20,000 g for 20 min. The supernatant was diluted four times with sodium acetate buffer and applied to a Bond Elute C18 cartridge (500 mg/6 ml, Harbor City, CA, USA) which has been pre-activated by 5-ml methanol. The cartridge was washed with 25% ethanol and eluted with 5-ml of methanol. Finally, the solvent was removed by gas flow (nitrogen gas), the residue was reconstituted with 1-ml of methanol. Precipitated solids were then removed by filtration using a 045-μm filter (Millipore, MA, USA). The BA extraction in the liver, appropriate modification methods were used and described by Yang et al. (21), ~50–60 mg of the lyophilized sample was homogenized in 1-ml physiological saline, the supernatant was obtained by centrifugation at 2,500 rpm for 10 min, 200-μl of liver homogenate was then extracted using the same method as described above. Finally, the resulting supernatant was used for LC-MS/MS analysis and the assay conditions have been reported previously (20). The BA in the intestinal contents was extracted in a similar manner as described by Tang et al. (13).

### Determination of mRNA Expression of Target Genes in the Ileum

The critical genes related to BA transportation, BA signaling, and antioxidant capacity were analyzed. Primers (Supplementary Table S3) were characterized by amplification efficiency analyses and agarose gel electrophoresis. Total RNA was isolated from the ileum using a total RNA extraction kit (Gene better, R013-50, Beijing, China). RNA integrity and concentration were assessed *via* 1.2% agarose gel electrophoresis and microspectrophotometer (Nanodrop, Technologies, Wilmington, DE, USA). First-strand cDNA was synthesized by using the High-Capacity cDNA Archive kit (Takara RR047A, China) according to the instructions of the manufacturer. Approximately 2-μg of RNA with an average A260/A280 of 1.9 were transcribed. The qPCR was performed on 384-well microplates and analyzed using ABI TaqMan 7900-HT software (Applied Biosystems, CA, USA). The comparative CT method (2^−ΔΔ^Ct) was used to calculate the gene expression values using β-*actin* as a housekeeping gene.

### 16S rRNA Gene Sequencing

Microbial community genomic DNA was extracted from cecal contents samples using the E.Z.N.A.^®^ soil DNA Kit (Omega Bio-Tek, Norcross, GA, USA) according to the instructions of the manufacturer. The DNA extract was checked on 1% agarose gel, and DNA concentration and purity were determined with NanoDrop 2000 UV-vis spectrophotometer (Thermo Scientific, Wilmington, USA). The hypervariable region V3–V4 of the bacterial 16S rRNA gene was amplified with 338F/806R primer pairs (338F, 5′-ACTCCTACGGGAGGCAGCAG-3′; 806R, 5′-GGACTACHVGGGTWTCTAAT-3′) by an ABI GeneAmp^®^ 9700 PCR thermocycler (ABI, CA, USA). The PCR amplification of 16S rRNA gene was performed as follows: initial denaturation at 95°C for 3 min, followed by 27-cycles of denaturing at 95°C for 30 s, annealing at 55°C for the 30 s and extension at 72°C for 45 s, and single extension at 72°C for 10 min, and end at 4°C. The PCR mixtures contain 5×TransStart FastPfu buffer (TransGen Biotech, Beijing, China) 4-μL, 2.5-mM dNTPs (Takara, Shiga, Japan) 2-μl, forward primer (5-μM) 0.8-μl, reverse primer (5-μM) 0.8-μl, TransStart FastPfu DNA Polymerase (TransGen Biotech, Beijing, China) 0.4 μl, template DNA 10 ng, and finally double-distilled water up to 20-μl. PCR reactions were performed in triplicate. The PCR product was extracted from 2% agarose gel and purified using the AxyPrep DNA Gel Extraction Kit (Axygen Biosciences, CA, USA) according to the instructions of the manufacturer and quantified using Quantus™ Fluorometer (Promega, USA). The raw 16S rRNA gene sequencing reads were demultiplexed, quality-filtered by fastp version 0.20.0 (Haplox, Shenzhen, China) (22), and merged by FLASH version 1.2.7 (Johns Hopkins University, Baltimore, MD) (23) with the following criteria: (1) the 300-bp reads were truncated at any site receiving an average quality score of < 20 over a 50 bp sliding window, and the truncated reads shorter than 50-bp were discarded, reads containing ambiguous characters were also discarded; (2) only overlapping sequences longer than 10-bp were assembled according to their overlapped sequence. The maximum mismatch ratio of the overlap region is 0.2. Reads that could not be assembled were discarded; (3) Samples were distinguished according to the barcode and primers, and the sequence direction was adjusted, exact barcode matching, two nucleotide mismatches in primer matching. Operational taxonomic units (OTUs) with 97% similarity cutoff (24, 25) were clustered using UPARSE software (version 7.1, http://drive5.com/uparse/) (24), and chimeric sequences were identified and removed. The taxonomy of each OTU representative sequence was analyzed by RDP Classifier version 2.2 (26) against the 16S rRNA database (e.g., Silva v138) using a confidence threshold of 0.7. The abundance data of representative sequences were normalized for each sample and were log-transformed.

### Statistical Analysis

The data of growth performance, serum and hepatic biochemical parameters, bacterial α-diversity, bile acids, intestinal morphometric indices, and mRNA expression levels of genes were analyzed by one-way ANOVA using the fitting model platform of JMP 10 software (SAS Institute, Inc., Cary, NC, USA). Statistical differences among the treatments were separated by the least significant difference method. Data were shown as the means with a standard error of the mean or standard error. Statistical significance was declared at a *P* < 0.05 and trends at 0.05 < *P* ≤ 0.10.

The statistical analysis of operational taxonomic unit (OTU) reads was performed using the R program (version 3.6.1, Vienna, Austria). For β-diversity analysis, principal coordinates analysis (PCoA) based on the Bray-Curtis distance matrices was used to visualize among groups. A permutational multivariate analysis of variance (PERMANOVA) was evaluated for microbial community structure comparison. Pairwise comparisons based on a negative binomial Wald test from the DESeq2 software package and sparse partial least squares discriminant analysis (sPLS-DA) from the mixOmics packages were used to measure the differences in individual OTUs at different taxon levels (27–29). A Benjamini and Hochberg-corrected *P*-value of 0.05 was considered statistically significant (30).

In order to identify critical features influencing the physiological responses to heat stress or exogenous bile acids, sparse partial least squares (sPLS) regression and relevance network analysis were performed using the mixOmics package (31, 32) in the R program to integrate the data sets of discriminant bacterial OTUs, bile acids, serum and liver biochemical parameters, as well as mRNA expression levels of BA-related genes. The threshold for absolute correlation was set to be at least 0.6.

## Results

### Growth Performance

As shown in Supplementary Table S1, there was no significant difference in the initial BW of all groups at d21 (*P* = 0.9640). And the effects of BA supplementation on the growth performance of heat-stressed birds were shown in Table 1, the average daily feed intake (ADFI) and average daily gain (ADG) of birds reared under 32°C were extremely significant lower than those in the thermoneutral environment (*P* < 0.0001). During the heat stress, the feed conversion ratio (FCR) of birds was greatly decreased from 0.56 to 0.48, while the ADG and FCR reductions were diminished by BA administration (*P* < 0.05). whereas the apparent digestibility of crude fat was not significantly affected by HS or BA supplementation (*P* > 0.05).

**Table 1:** Effect of BA on the growth performance of heat-stressed broiler chickens.

	**Groups**			***P*** **-value**
	**TN**	**HS**	**HS-BA**	
ADFI, g	147.92 ± 0.73^a^	110.25 ± 1.17^b^	110.27 ± 3.46^b^	<0.0001
ADG, g	80.38 ± 0.48^a^	49.76 ± 0.90^c^	54.68 ± 1.98^b^	<0.0001
FCR	0.56 ± 0.01^a^	0.48 ± 0.02^c^	0.51 ± 0.02^b^	<0.0001
Digestibility of CF, %	84.00 ± 1.08	83.94 ± 2.47	85.34 ± 1.76	0.3621

*All data are expressed as M ± SD (n = 6 per group). In the same row, values with different small letter superscripts mean a significant difference (P < 0.05). ADFI, average daily feed intake; ADG, average daily gain; FCR, feed conversion ratio; Digestibility of CF, apparent digestibility of crude fat*.

### Antioxidant Activity in Serum and Liver

As shown in Table 2, HS significantly decreased GSH-Px activities in the serum (*P* = 0.049) and liver (*P* = 0.002), which was reversed after treatment with BA. However, MDA (biomarkers for tissue oxidative damages) and SOD activities were not significantly changed in both serum and liver (*P* > 0.05) by the increased environmental temperature or BA supplementation. Besides, HS and exogenous BA increased the expression of *Keap-1* (*P* = 0.0104) without the impact on *Nrf-2* expression (**Figure 6I**).

**Table 2:** Oxidative damage parameters and antioxidative enzymes in the serum and liver.

		**Groups**			***P*** **-value**
		**TN**	**HS**	**HS-BA**	
Serum	MDA, nmol/mL	1.19 ± 0.09	1.80 ± 0.56	1.26 ± 0.10	0.3877
	SOD, U/mL	20.42 ± 0.46	21.44 ± 0.44	20.01 ± 0.78	0.2356
	GSH-Px, U/mL	1219.82 ± 93.20^a^	788.26 ± 128.78^b^	1216.88 ± 156.09^a^	0.0485
Liver	MDA, nmol/g	2.62 ± 0.44	3.56 ± 0.56	2.96 ± 0.16	0.3064
	SOD, U/g	330.05 ± 39.18	339.10 ± 34.92	291.16 ± 24.53	0.5719
	GSH-Px, U/g	1233.79 ± 52.40^a^	896.40 ± 52.98^b^	1301.37 ± 95.08^a^	0.0020

*All data are expressed as M ± SD (n = 6 per group). In the same row, values with different small letter superscripts mean a significant difference (P < 0.05). MDA, malondialdehyde; SOD, superoxide dismutase; GSH-Px, glutathione peroxidase*.

### Spatial Heterogeneity of BA Profiles in Broiler Biological Samples

**Figure 1:**
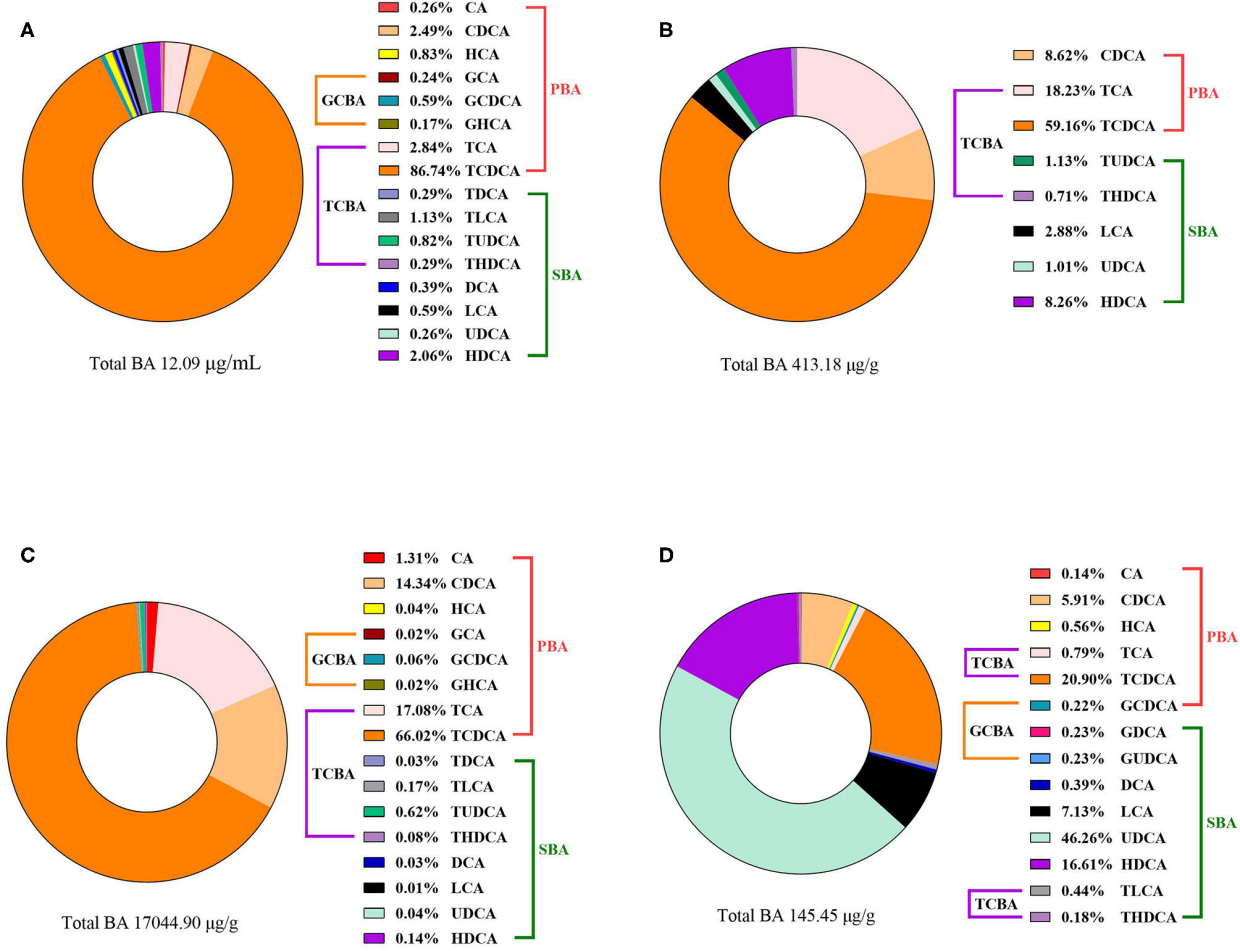
The compositions of BA in the **(A)** serum, **(B)** liver, **(C)** ileal contents, and **(D)** cecal contents of broiler chickens in the TN group. Data of total BA are expressed as mean value, and each BA was calculated as % of total BA (*n* = 6).

Bile acid concentrations and composition exhibited large differences in various compartments as shown in Figure 1. In the present study, analysis of the BA profiles revealed BA in broiler chickens is primarily conjugated with taurine, accounting for the majority of total-BA in the serum, liver, ileum, and cecum. In the serum, taurochenodeoxycholic acid (TCDCA) was present in a ratio of 86.74%, and the taurine to glycine bile acids ratio was ~92:1 in the BA pool. The liver is the critical site for BA synthetic regulation, most of BA was conjugated with taurine, Tauro-BA (TCBA) such as TCDCA (59.16%) and TCA (18.23%) played a leading role, which constituted more than 77% of total BA in the liver. Similar results of the BA composition were observed for serum and hepatic, TCDCA (66.02%), TCA (17.08%), and CDCA (14.34%) were still the predominant BA in ileal contents. However, through a BA quantification in the intestine contents of healthy birds, we found that the total-BA in the cecal contents merely accounted for 0.83% of those in the ileum, because of absorption of BA in the terminal ileum, and 70.75% of total-BA were secondary BA (SBA) in the cecum.

### The Alterations in BA Profiles, BA Transporter, and Signaling Caused by HS and HS-BA Treatments

**Figure 2:**
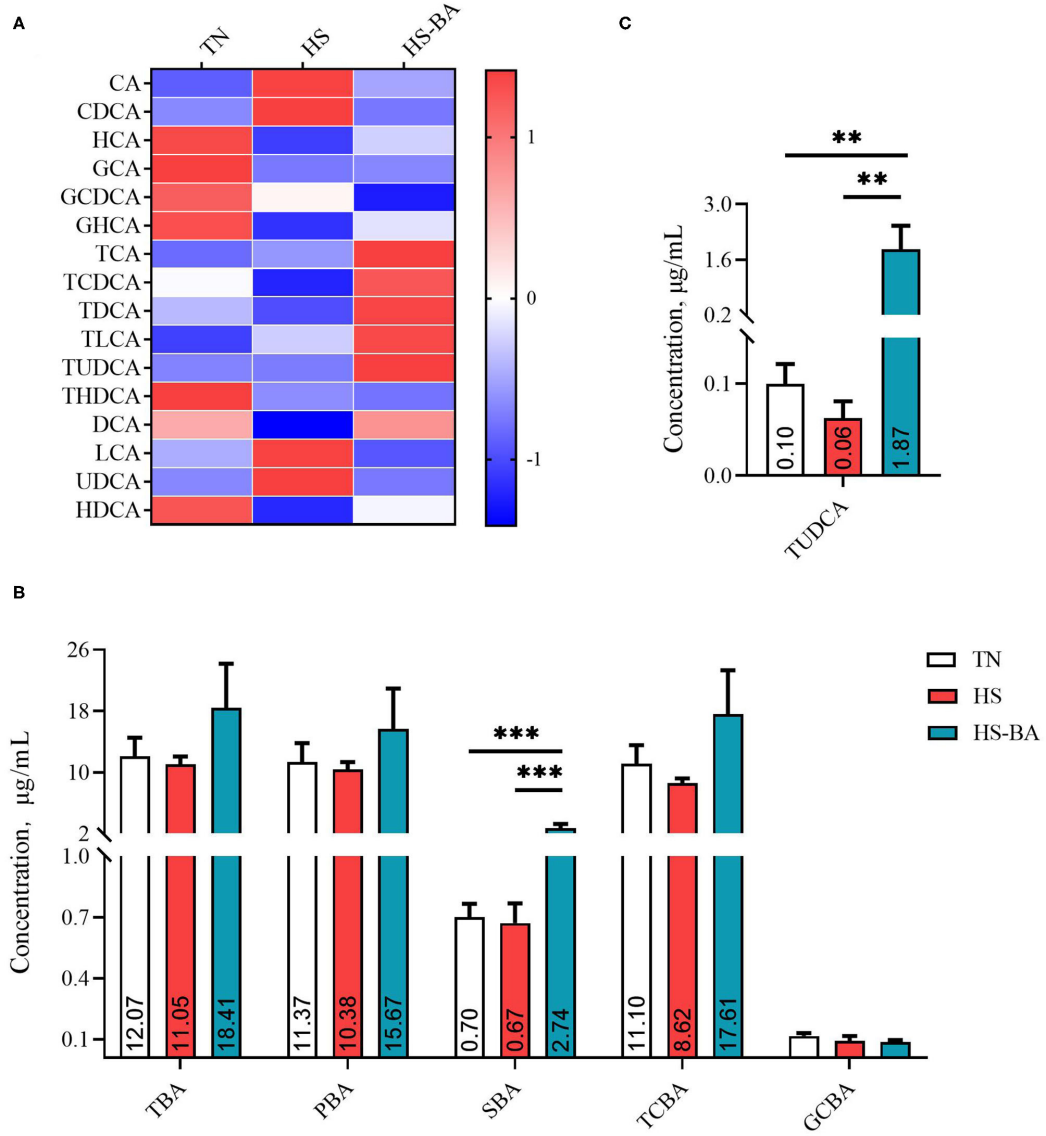
The alteration in serum BA profiles of broiler chickens caused by HS, or by HS-BA treatment. **(A)** The heatmap shows the BA profiles of each group (data were represented as the *Z*-score, the same below); **(B)** each type of BA content, and **(C)** the significant differences in individual BA content were shown and marked. Data were presented with mean ± SEM (*n* = 6). ^**^P < 0.01; ^***^P < 0.0001.

As illustrated by Figure 2B, dietary BA supplementation significantly increased the serum SBA level (*P* = 0.0003). Among them, supplementing BA in the diet increased significantly the TUDCA level (*P* = 0.0026). Whereas, it was found to have no significant effect on TBA, PBA, TCBA, and GCBA in serum.

**Figure 3:**
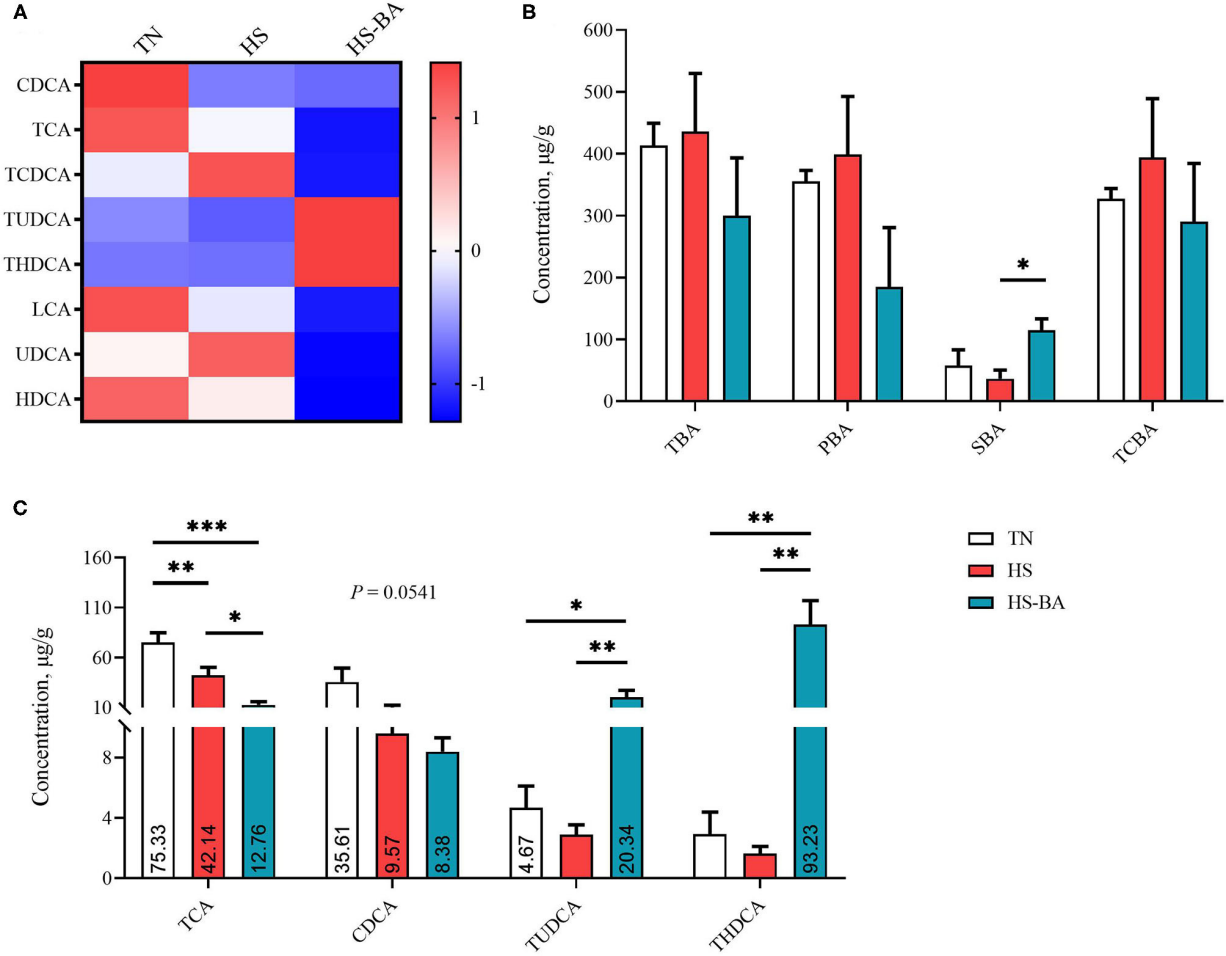
The alteration in liver BA profiles of broiler chickens caused by HS, or by HS-BA treatment. **(A)** The heatmap shows the BA profiles of each group; **(B)** each type of BA content, and **(C)** the significant differences in individual BA content were shown and marked. Data were presented with mean ± SEM (*n* = 6). ^*^P < 0.05; ^**^P < 0.01; ^***^P < 0.0001.

For the alteration of BA profiles in the liver (Figure 3), SBA was lower in the HS group than those in the TN group (−36.88%) and was significantly lower than those in the HS-BA group (*P* = 0.0368). However, birds in the BA group exhibited significantly higher liver TUDCA (*P* = 0.0154) and THDCA (*P* = 0.0003) levels than those in the TN and HS group. HS (*P* = 0.0062) and exogenous BA (*P* < 0.0001) resulted in decreased TCA and tended to decreased CDCA in the liver (*P* = 0.0541).

**Figure 4:**
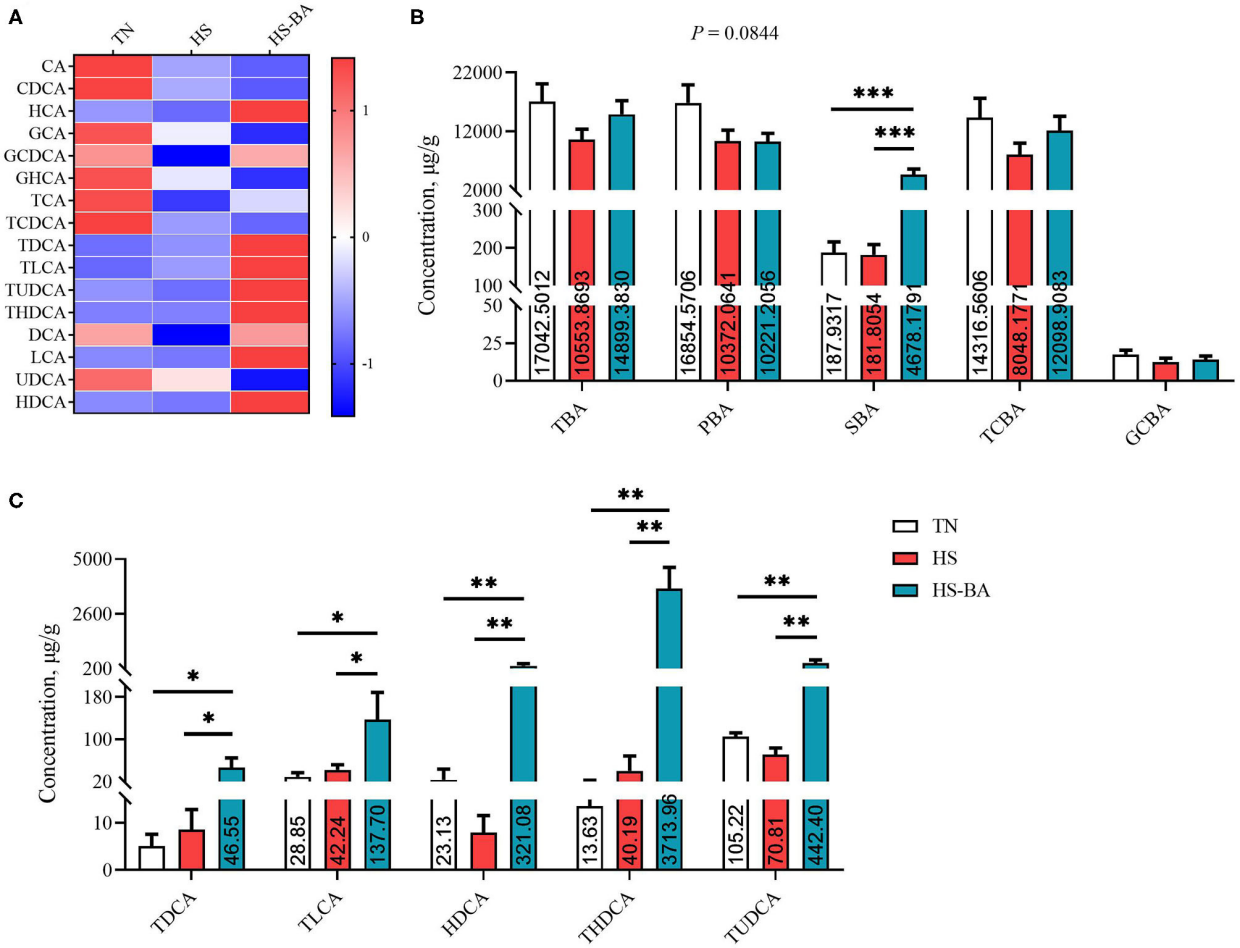
The alteration BA profiles in ileal contents of broiler chickens caused by HS, or by HS-BA treatment. **(A)** The heatmap shows the BA profiles of each group; **(B)** each type of BA content, and **(C)** the significant differences in individual BA content were shown and marked. Data were presented with mean ± SEM (*n* = 6). ^*^P < 0.05; ^**^P < 0.01; ^***^P < 0.0001.

Under the HS and exogenous BA conditions, there was a trend toward lower PBA in the ileal contents (*P* = 0.084) by 38.46 and 39.36% (Figure 4). The ileal contents of the BA group had higher levels of SBA than the HS and TN group (*P* < 0.0001), but the low TCBA level was not significantly different between these groups. BA supplementation indeed increased TDCA (*P* = 0.0307), TLCA (*P* = 0.0453), HDCA (*P* = 0.0018), THDCA (*P* = 0.0002), and TUDCA (*P* = 0.0072) levels.

**Figure 5:**
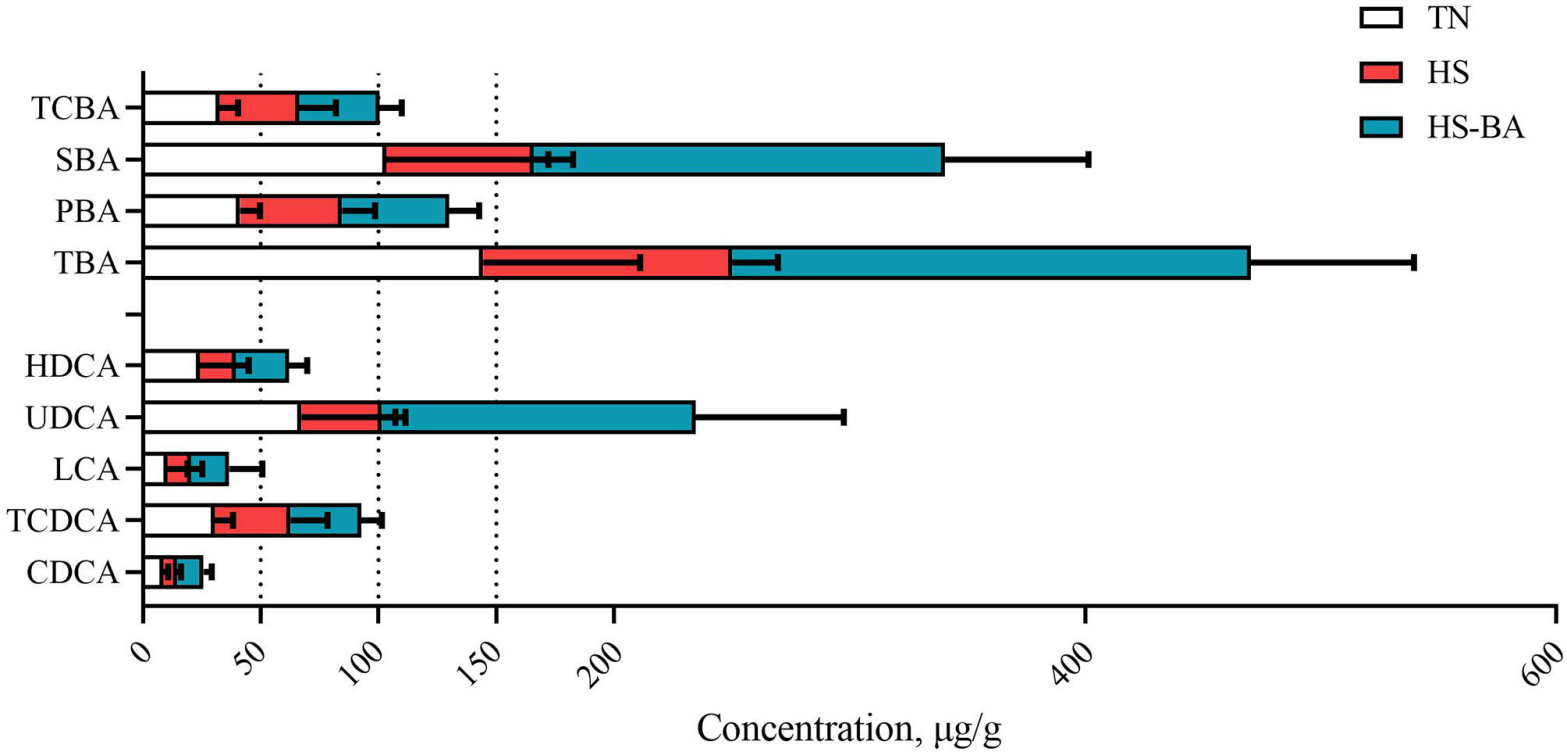
The alteration of BA profiles in cecal contents of broiler chickens caused by HS, or by HS-BA treatment. Each type of BA content and the main individual BA content were shown. Data were presented with mean ± SEM (*n* = 6).

It could be seen from Figures 2–4 that BA supplementation and exposure to heat were significantly affected the PBA, SBA, or TCBA levels in the serum, liver, and ileal contents. Whereas, no significant change was observed in the cecal contents BA (Figure 5), only HS-caused SBA was decreased by 39.45% compared with the TN group, but there is no statistical difference between groups (*P* > 0.05).

**Figure 6:**
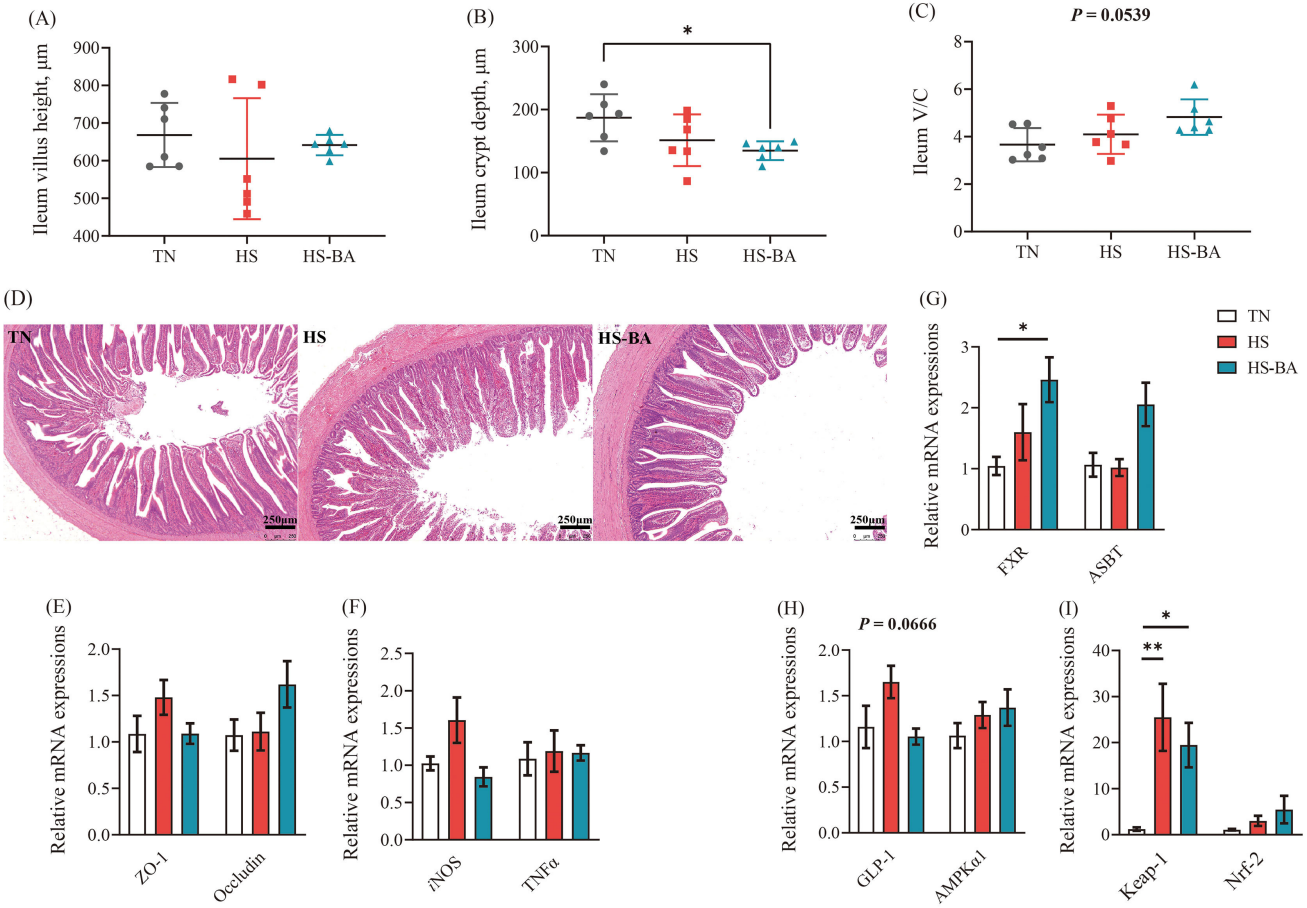
Effects of exogenous BA on the intestinal morphology and the mRNA expression levels of tight junction, inflammatory cytokines, as well as BA-related genes in ileum of heat-stressed broiler Chickens. **(A–C)** The villus height, crypt depth and V/C of ileum. Tissue sections of intestinal mucosa in ileum [Scale bars: 250 microns, (**D**)]. Protein expressions of ZO-1, Occludin, *i*NOS, TNFα, FXR, ASBT, GLP-1, AMPKα1, Keap-1, and Nrf-2 in the ileum **(E–I)**. Data were the means ± SEM (*n* = 6 birds). * indicates *P* < 0.05 and ** indicates *P* < 0.01. AMPKα1: adenosine 5′-monophosphate (AMP)-activated protein kinase-α1, ASBT: the apical ileal sodium-dependent bile acid cotransporter, FXR: farnesoid x receptor, GLP-1: glucagon-like peptide 1, *i*NOS: inducible NO synthase, Keap-1: kelch-like ECH-associated protein 1, Nrf2: nuclear factor-like 2, ZO-1: zonula occludens-1, TNFα: tumor necrosis factor-α.

As for the transportation of BA in the ileum, the expression of *FXR* (*P* = 0.0369) and *ASBT* (*P* = 0.0154) were increased in the HS-BA group (Figure 6G). Moreover, we also observed that HS tended to increase the *GLP-1* expression (*P* = 0.067) (Figure 6H).

### Intestinal Morphology and mRNA Expression Levels of Tight Junction Proteins as Well as Inflammatory Cytokines in the Ileum

As illustrated by H&E staining of the ileum and its measurement parameters (Figures 6A–D), birds fed with BA supplementation reduced (*P* = 0.0431) crypt depth, and the ratio of villous height to crypt depth trended higher (*P* = 0.0539) under the heat exposure. The ileum villi were partly exfoliated in the HS group (heat exposure for 21 days). Even so, the villus structure in the HS-BA group remained relatively intact. However, HS and exogenous BA did not affect the expression of *ZO-1* and *Occludin* (*P* > 0.05) (Figure 6E). On the other hand, BA significantly decreased *iNOS* mRNA level (*P* = 0.0399) but had no obvious effect on *TNF*α mRNA level compared with the HS group in the ileum (Figure 6F).

### Bacterial Community Profiles

Heat stress-caused SBA was decreased in the liver and cecum by 36.88 and 39.45%, respectively. We further examined whether exposure to heat caused changes to the intestinal environment. Before calculating α- and β-diversity, samples were rarefied to 28,286 reads to account for unequal numbers of sequences among samples. After filtration for the rare OTU, a total of 1,059 OTU remained in our data set. The species richness estimators (Chao1), evenness (Simpson and ACE index), and diversity index (Shannon) were comparable among all treatments in the cecum (Supplementary Figures S1A–D), and there was no difference in microbial α-diversity. Based on Bray-Curtis dissimilarity matrices at the OUT level, the cecal samples in the HS group exhibited a distinct cluster that was clearly separated from those in the TN group (R^2^ =0.18, *P* < 0.03) (Supplementary Figure S1E).

**Figure 7:**
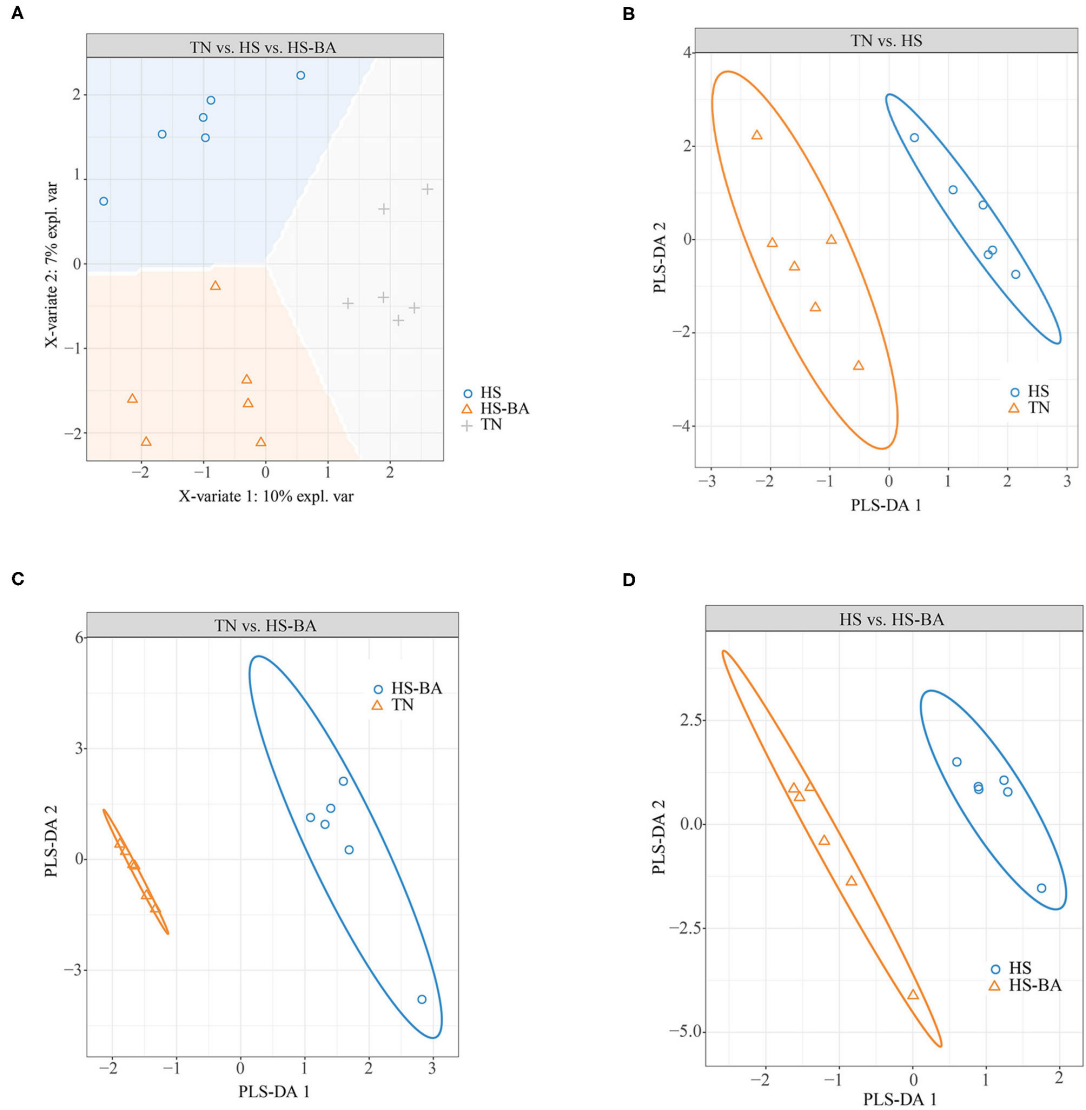
Score plot of 2-component sparse partial least square discriminant analysis models showing gut microbiota clustering according to the environment in the cecum of birds under HS or HS-BA treatments **(A–D)** with the percentage of variance captured for each principal component.

**Figure 8:**
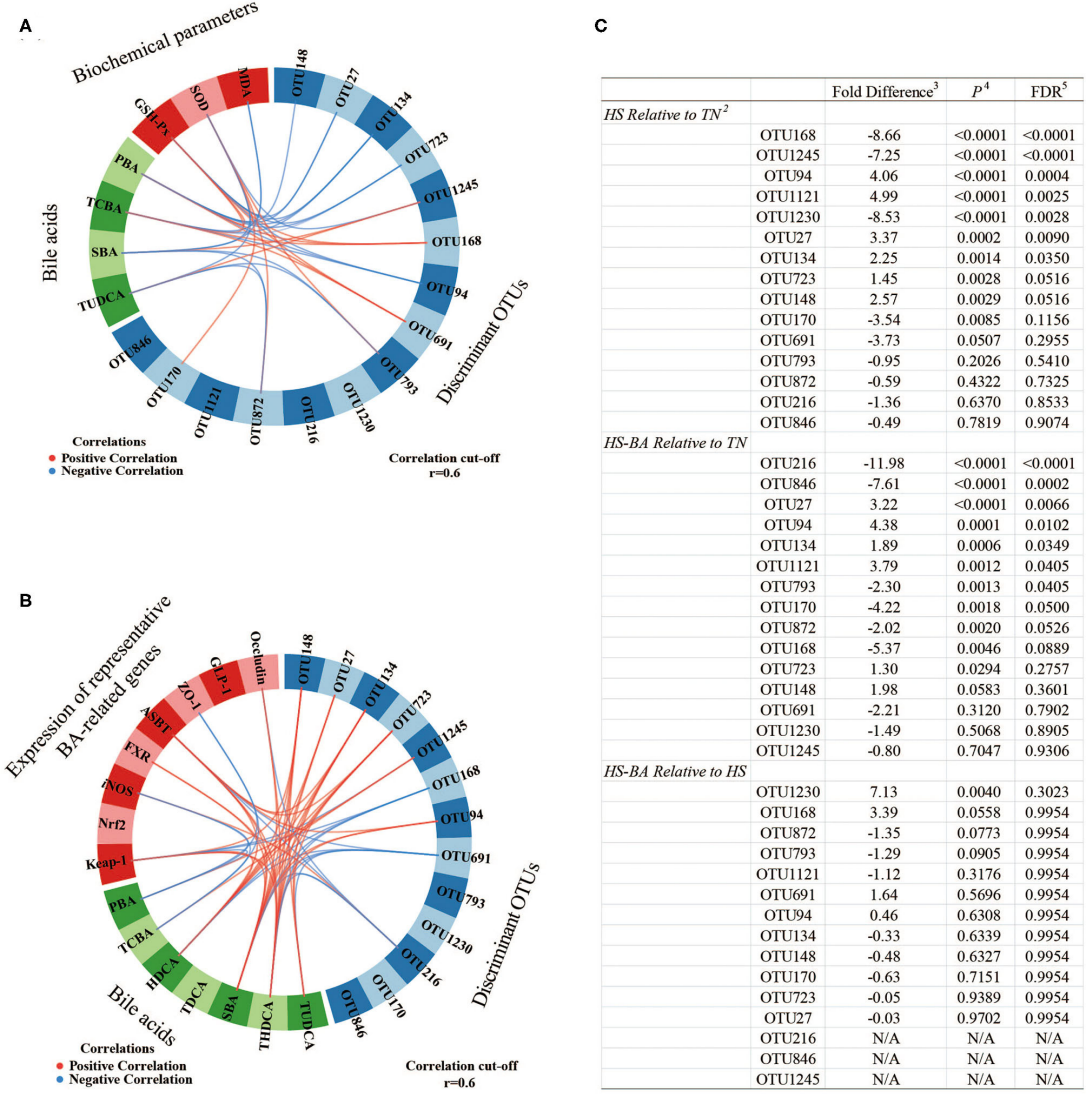
The circos plots of sparse partial least square-discriminant analysis displaying correlations between the identified best discriminant bacterial operational taxonomic units 1 (OTU; *n* = 15; relative OTU abundance > 1%), expression levels of target genes, serum biochemical parameters, and BA. Positive and negative correlations (|r| > 0.6 for the component) are displayed by red and blue links, respectively. OTU168, *unclassified Streptococcus*; OTU1245, *gut metagenome Alistipes*; OTU94, *Alistipes inops*; OTU1121, uncultured Merdibacter; OTU1230, *Lactobacillus johnsonii*; OTU27, *unclassified Christensenellaceae R-7 group*; OTU134, uncultured *Alistipes*; OTU723, *Alistipes inops*; OTU148, unclassified Ruminococcaceae; OTU170, *unclassified Turicibacter*; OTU691, *gut metagenome Alistipes*; OTU793, uncultured Oscillospiraceae; OTU872, unclassified Ruminococcaceae; OTU216, *Barnesiella viscericola DSM 18177*; OTU846, uncultured Fournierella. 1. Pairwise comparison of diet group using Negative Binomial Wald Test. 2. Taxonomy assignments based on Silva Database. 3. Log2 fold differences. 4. *P*-value. 5. Benjamini and Hochberg FDR correction.

Overall, the flora in the cecal niche of broilers is mainly composed of Firmicutes, Bacteroidota, Proteobacteria, and Actinobacteria (Supplementary Figure S1F). Heat-stressed broilers which fed with a Basal diet displayed an increased abundance of Firmicutes (59.6 vs. 48.02%) and decreased abundance of Bacteroidota (38.81 vs. 47.80%). A core bacterial community was determined based on the DESeq2 method and sPLS-DA analysis implemented in R programming software (Figure 7). As indicated in Figure 8C, a total of 15 most discriminant bacterial OTUs among the treatment groups were identified in the cecum.

### Associations Between Cecal OTUs, BA, Biochemical Parameters, and BA-Related Genes in the Host

The sPLS regression offered us a potentially effective model to classify the most discriminant bacterial OTUs, serum biochemical parameters, BA, and BA-related genes (Figure 8). For example, unclassified *Streptococcus*-OTU168 and *gut metagenome Alistipes*-OTU691 abundances were positively correlated to serum TCBA concentration. In contrast, *Alistipes inops*-OTU94 and uncultured *Alistipes-*OTU134 abundances were positively correlated to serum TCBA concentration, while uncultured Oscillospiraceae-OTU793 and unclassified Ruminococcaceae-OTU872 were negatively associated with serum TUDCA and even SBA concentrations. And *gut metagenome Alistipes*-OTU1245 was positively correlated to SBA and TUDCA in the serum and ileum. The abundance of unclassified *Christensenellaceae R-7 group*-OTU27 and *Alistipes inops*-OTU723 were positively correlated to serum TUDCA and THDCA concentrations. Furthermore, three OTUs (unclassified *Streptococcus-*OTU168, *gut metagenome Alistipes*-OTU691, and *Barnesiella viscericola DSM 18177*-OTU216) were negatively associated with ileum SBA such as THDCA. Whereas, four OTUs (*Christensenellaceae R-7 group*-OTU27, uncultured *Alistipes-*OTU134, *Alistipes inops*-OTU723, and unclassified Ruminococcaceae-OTU148) were positively correlated to ileum SBA such as HDCA and THDCA concentrations. For the relationships between BA, serum biochemical parameters, and the mRNA expression levels of BA-related genes, the TCBA was positively correlated to GSH-Px activity but it correlated negatively with MDA level in the serum. In addition, SBA such as THDCA was positively correlated to genes of *Occludin, ASBT, FXR*, and *Keap-1*, meanwhile, PBA and TCBA were negatively associated with genes of *ZO-1* and *iNOS* in the ileum.

## Discussion

The high ambient temperature has a negative effect on the growth performance of broiler chickens in the middle and later stages, and adversely impacts physiological parameters by inducing oxidative stress as well as abnormal lipid metabolism (4, 5, 33). A decreased feed intake is the most common and intuitive impact of HS on broilers. Consequently, the growth performance of broilers cannot be guaranteed, where ADG is decreased by 38% under the HS. Emerging evidence has demonstrated a strong correlation between oxidative stress, growth performance, and metabolic disorders (34). High-intensity HS induces intestinal ischemia, as well as autolysis and sloughing the intestinal villi, leading to impairments of intestinal morphology, or reduction of antioxidant enzyme activities (35, 36). Misregulation in this ROS-scavenging process leads to the generation of excessive amounts of ROS, it can damage lipids, proteins, and DNA, which is associated with changes in mitochondrial functions (37–39). In the present study, however, these heat-stressed birds did not develop obvious oxidative damages at 32°C, as there were no significant changes in serum and liver MDA, but HS significantly decreased GSH-Px activities in serum and liver of heat-stressed birds. Notably, it was collectively consistent with the TUDCA content was significantly increased in the serum and liver, and ileum by supplementing the BA in the diet. Hydrophilic BA like (Tauro-) UDCA as the classic endoplasmic reticulum (ER) stress inhibitor, could alleviate ER stress and restore glucose homeostasis (40). Paumgartner and Beuers indicated that TUDCA is a nutritional preventive strategy in alleviating oxidative stress and protecting cells (41). Furthermore, previous research has also indicated that (Tauro-) UDCA could be used as a molecular chaperone to enhance protein folding to increase the level of glutathione in the liver, and protect liver Huh7 cells against TG induced morphologic changes of endoplasmic reticulum stress and apoptosis (42, 43). Indeed, BA supplementation ameliorated the decreased GSH-Px activities in the serum and liver in heat-stressed birds. It has also been shown that heat exposure decreased villi height and the ratio of villi height to crypt depth in the ileum of broilers as compared to those in control and pair-fed groups, which is not conducive to the digestion and absorption of nutrients (44, 45). However, heat-stressed birds fed with BA supplementation had a trend toward a higher ratio of villi height to crypt depth in the ileum. On the other hand, the micellization process of BA increases intraluminal lipids in the surface area and improves the accessibility of intestinal lipase and the efficiency of fat hydrolysis (46). Therefore, BA was expected to improve growth performance by facilitating fat digestion. Previous two studies showed that dietary supplementation with 60~80 mg/kg BA compound for 42-d improved the performance of broilers by elevating the activities of lipoprotein lipase and duodenum lipase (7, 47). In the current study, heat-stressed birds fed with a BA-supplemented diet increased the ADG by about 10% with a significant effect on feed conversion ratio (from 0.48 to 0.51), however, the apparent digestibility of crude fat (1.4% increase). Although the exogenous BA did not seem to improve the digestion of fat, the BA supplementation contributed to a potential way to promote the growth of birds under the HS. Besides, our results showed that heat stress up-regulated the expression of ileal kelch-like ECH-associated protein 1 (*keap-1*), and regardless of TN or HS, and exogenous BA similarly up-regulated the expression of keap-1 in ileal. Inactive Nrf-2 is bound to its suppressor Keap-1 and sequestered in the cytoplasm under normal conditions (48). The expressions of *Nrf-2* and antioxidant enzymes were not altered when the temperature or diet alternated. Varasteh et al. reported that *i*NOS level was negatively associated with intestinal integrity, an increase of *i*NOS level seems to represent a late inflammatory response (49). And a decreased expression of *iNOS* mRNA in the ileum was observed in heat-stressed birds fed with BA supplementation.

To the best of our knowledge, it is the first study to profile BA composition in compartments to provide a comprehensive investigation of whether HS alters BA compositions and the availability of exogenous BA in heat-stressed chickens. By evaluating the compositions of BA profiles throughout the enterohepatic circulation, we obtained a biogeographical view of BA-mediated ecological impact. And the enterohepatic circulation of BA exerts important physiological functions not only in the digestion of lipids and fat-soluble vitamins but also in control of whole-body lipid homeostasis (50). The previous study has indicated that mild HS caused hepatic lipid accumulation, whereas dietary supplementation of BA decreased triglycerides and the expressions of *SREBP-1c* and *FAS* (51). Unlike the growing pigs that were exposed to high ambient temperatures, heat exposure did not suppress BA synthesis of broiler in our previous report, however, the results indicated that the supplementation of BA was necessary under the HS (6, 52). Research suggested that metabolomics provides evidence points to BA as signature metabolites for heat stress (53). The decrease of CA, DCA, and even TCBA was observed in the plasma of rats after 48-h of acute HS (54). Here we noted that hepatic and cecal SBA was decreased in birds exposed to 32°C, and the same trend was observed for PBA (mainly TCDCA) in the ileum. (Taurine-) CDCA, the primary bile acid of LCA and UDCA, have stronger ligand activity to FXR than LCA and DCA. In mice, it has been reported that CDCA supplementation restored LPS-induced elevation of intestinal permeability and MLCK expression and reduction of tight junction protein expression, thus alleviating LPS-induced intestinal barrier impairment (55). Dietary CDCA improves the growth performance of weaned piglets by improving intestinal morphology and barrier function and enhancing lipid digestion. Nevertheless, cross-species function studies are inconsistent, the side effects of the single BA supplementation can be problematic. Piekarski et al. reported that chickens fed with diet-containing 0.1% or 0.5% CDCA for 2 weeks exhibited a significant and dose-dependent reduction of feed intake and body weight by modulating the expression of appetite-related hypothalamic neuropeptides (56). The proportion of Tauro-CDCA in the ileum was 66.02% and this reduction by HS was probably an adaptive strategy of the cells in response to sophisticated environments. And the 7α-hydroxyl groups in CDCA can be epimerized to 7β from ursodeoxycholic acid (UDCA). Hydroxylation at the 6α/β or 7β-position increases solubility and reduces the toxicity of bile acids (9). Moreover, Perez and Briz indicated that UDCA and TUDCA can induce the expression of *CCK* in intestinal epithelial cells, and promote the immune regulation and bile secretion of hepatocytes and bile duct epithelial cells (43). Additionally, BA is reabsorbed *via* ASBT-mediated active transport or passive transport (57). In the ileum, we noted that an increase in the expression of *ASBT* and *FXR* after supplementing BA. Exogenous BA supplementation resulted in increases of several BA species, namely TUDCA, HDCA, and THDCA in the ileum, which can also function as a weak FXR agonist (58). However, the conflicting results of FXR induce *ASBT* expression in mice and inhibit *ASBT* expression in rabbits but do not affect ASBT in humans (59). Moreover, it was reported that GLP-1 regulated by FXR, and inhibited gastric emptying and suppressed food intake in both broiler and layer chicks (60). Exogenous BA inhibited the expression of *GLP-1* by up-regulating the expression of ileal *FXR* in heat-stressed broilers, thus can regulate and altering host physiologic responses (61). Relevant research has provided supporting evidence that HCA species (HCA, HDCA, and THDCA, etc.) are protective against the development of diabetes in mammals and have the potential to be used as a treatment for type 2 diabetes (62). We noted that the increases of HDCA and THDCA in the gut-liver axis after supplementing BA, besides its role in lipid emulsification, whether HCA species beneficial or harmful in heat-stressed broilers needs further investigation.

It has been indicated that the BA and microbiota could be considered an important role in gastrointestinal health (63, 64). Bile acids are produced in the liver as primary BA and metabolized in the gut to secondary BA, which participates in the microbiota modifications (deconjugation and dehydroxylation) with *Bacteroides, Clostridium, Eubacterium, Lactobacillus*, and *Escherichia* (65, 66). However, a previous report indicated that HS disturbed the stabilization of intestinal microbial ecology, it decreased the viable counts of *Lactobacillus* and *Bifidobacterium* and increased the viable counts of *coliforms* and *Clostridium* in the small intestine (67). HS also altered the cecal microflora profile of broilers, which displayed the increasing relative abundances of the phylum Firmicutes and the genus *Tyzzerella* while the relative abundances of the phylum Bacteroidetes, the genera *Bacteroides, Parabacteroides*, and *Romboutsia* were decreased (68). Similarly, HS mediated alterations in the gut microbial community structures were observed in the cecum, members of the Firmicutes and Bacteroidota contributed to the alterations in the cecum. Accordingly, there was an obvious difference between HS and TN from the PcoA plot in the cecum, the abundances of most discriminant bacterial OTUs among the treatment groups based on sPLS-DA analysis were found to be significantly influenced by HS compared to the TN group. In contrast, few specific OTUs were different due to dissimilar diets under HS conditions. Among these, the decrease in the abundance of *unclassified Streptococcus, unclassified Turicibacter, gut metagenome Alistipes, Lactobacillus johnsonii* under HS condition were observed. While the microbiota resident in the gut is now known to provide a range of functions relevant to host health, many of the microbial members of the community have not yet been cultured or classified. For example, our results indicated that a positive association between *gut metagenome Alistipes* and *unclassified Streptococcus* and TCBA or SBA levels in serum may associate inhibition of BA conversion with imbalanced gut microbiota induced by HS. Well-known probiotics such as *Lactobacillus johnsonii* appearing to be the most acid-resistant strain are thought to be beneficial to the areas of immunomodulation, pathogen inhibition, and cell attachment (69). Moreover, some *Turicibacter* species, which has been strongly associated with immune function and bowel disease, indicating that may have a favorable effect on gut health (70). In our research, mild heat stress (32°C) seems to have little effect on harmful bacteria multiplication. However, Tsiouris et al. reported that cyclic acute HS (35°C) affected the cecal *Clostridium perfringens* counts in broilers, resulting in necrotic enteritis (71). It was likely the intestinal flora is more sensitive to high ambient temperature than the duration of HS. Naturally, exogenous BA then enters the small intestine quickly after oral administration, and most Bas are absorbed in the terminal ileum. The circos plots (Figure 8B) showed that SBA such as THDCA has a positive correlation with expressions of *FXR, occludin, ASBT*, and *Keap-1* while the PBA and TCBA were negative correlation with expressions of *ZO-1* and *iNOS* in the ileum, indicating that the functions of exogenous BA are diverse and complex. Note that since the hydrophobicity of BA determines its toxicity and protective effect, the profile of the exogenous BA can greatly affect its function (72). Further comprehensive nutritional, physiological, microbiome, genomics, and metabolomics measurements associated with exogenous BA are warranted to accurately determine the mode of action, and indeed whether any growth promotion in animal husbandry can be achieved *via* healthy approaches.

In fact, our trial also has a limitation in that it lacks the pair-fed group. Despite this limitation, several studies are using the pair-fed treatment based on the same feed intake to determine whether heat exposure directly or indirectly (*via* reduced feed intake) causes these negative impacts in broiler chickens. Compared with pair-fed birds exposed to thermal neutral, heat-stressed chickens still exhibit slower growth and decreased feed efficiency (44). Similar results were observed in another study. HS caused the negative energy balance and broilers unable to effectively mobilize fat, thereby resulting in protein decomposition, which subsequently affected growth performance and carcass characteristics (73). Also, the alterations of amino acids, BA, and NEFA levels in serum were observed (74).

## Conclusion

Overall, our results showed that heat stress affected the modification of primary bile acids by altering gut microbiota composition in broiler chickens, leading to disturbance of bile acid metabolism, which probably influenced their heat stress resistance capability. Bile acids such as CA, CDCA, and HCAs have a positive effect on relieving abnormal lipid metabolism induced by heat stress has been reported, and the benefits of the dietary supplementation of bile acids contributing to the heat-stressed broilers are improvement in their average daily gain, the feed conversion ratio, and antioxidant capacity, indicating that bile acid as a nutritional strategy has a certain potential in alleviating heat stress. Furthermore, some bile acids such as tauro-conjugated UDCA and HCAs may play important roles in maintaining homeostasis in the gut-liver axis. However, more work needs to be done to better understand the molecular mechanisms of these bile acids in livestock and poultry applied research.

## Data Availability Statement

The data used to support the findings of this study are available from the corresponding author upon request. The raw data on cecal microbiota of broiler chickens were deposited in NCBI's Sequence Read Archive (SRA) database and accessible through SRA accession number: PRJNA750333.

## Ethics Statement

The animal study was reviewed and approved by the Experimental Animal Welfare and Ethical Committee of the Institute of Animal Sciences, Chinese Academy of Agricultural Sciences.

## Author Contributions

CY, RZ, LC, and HZ participated in the conception and design of the experiment. CY carried out the animal experiment with the help of BX, ST, and LL. BA compositions of BA supplementation and samples were measured and analyzed by BX and AC. CY wrote and modified the manuscript, and then BX, RZ, and LC reviewed and modified the final manuscript. All authors agree to be accountable for the content of the work.

## Funding

This research was supported by the National Key Research and Development Program of China (2016YFD0500501), Agricultural Research Outstanding Talents and Innovation Team (2016-nybrc-03), the Fundamental Research Funds for the Central Institute, and the Agricultural Science and Technology Innovation Program (ASTIP-IAS07).

## Conflict of Interest

AC was employed by Shandong Longchang Animal Health Care Co., Ltd. The remaining authors declare that the research was conducted in the absence of any commercial or financial relationships that could be construed as a potential conflict of interest. The handling Editor declared a shared affiliation with the authors at time of review.

## Publisher's Note

All claims expressed in this article are solely those of the authors and do not necessarily represent those of their affiliated organizations, or those of the publisher, the editors and the reviewers. Any product that may be evaluated in this article, or claim that may be made by its manufacturer, is not guaranteed or endorsed by the publisher.

## Abbreviations

ASBT: the apical ileal sodium-dependent bile acid cotransporter
ADG: average daily gain
AMPKα1: adenosine 5′-monophosphate (AMP)-activated protein kinase-α1
BA: bile acids
CA: cholic acid
CDCA: chenodeoxycholic acid
DCA: deoxycholic acid
FDR: false discovery rate
FXR: farnesoid x receptor
GCA: glycol-cholic acid
GCBA: glyco-conjugated bile acids
GCDCA: glycol-chenodeoxycholic acid
GHCA: glycol-hyocholic acid
GLP-1: glucagon-like peptide 1
GSH-Px: glutathione peroxidase
GUDCA: glycol-ursodeoxycholic acid
HCA: hyocholic acid
HDCA: hyodeoxycholic acid
HS: heat stress
*i*NOS: inducible NO synthase
Keap-1: kelch-like ECH-associated protein 1
LCA: lithocholic acid
LPS: lipopolysaccharide
MDA: malondialdehyde
Nrf2: nuclear factor-like 2
OTU: operational taxonomic unit
PBA: primary bile acids
PCoA: principal coordinate analysis
SBA: secondary bile acids
SCFA: short-chain fatty acid
SOD: superoxide dismutase
TBA: total bile acids
TCA: tauro-cholic acid
TCBA: tauro-conjugated bile acids
TCDCA: tauro-chenodeoxycholic acid
TDCA: tauro-deoxycholic acid
THDCA: tauro-hyodeoxycholic acid
TLCA: tauro-lithocholic acid
TN: thermal neutral
TNFα: tumor necrosis factor-α
TUDCA: tauro-ursodeoxycholic acid
UDCA: ursodeoxycholic acid
ZO-1: zonula occludens-1.
