# Supplementary material for: The Effect of Exogenous Bile Acids on Antioxidant Status and Gut Microbiota in Heat-Stressed Broiler Chickens
Source: Front Nutr. 2021 Nov 24;8:747136. doi: 10.3389/fnut.2021.747136 (PMC8652638; doi:10.3389/fnut.2021.747136)
Supplement: Supplementary file 1 [file Data_Sheet_1.PDF]

Supplementary materials

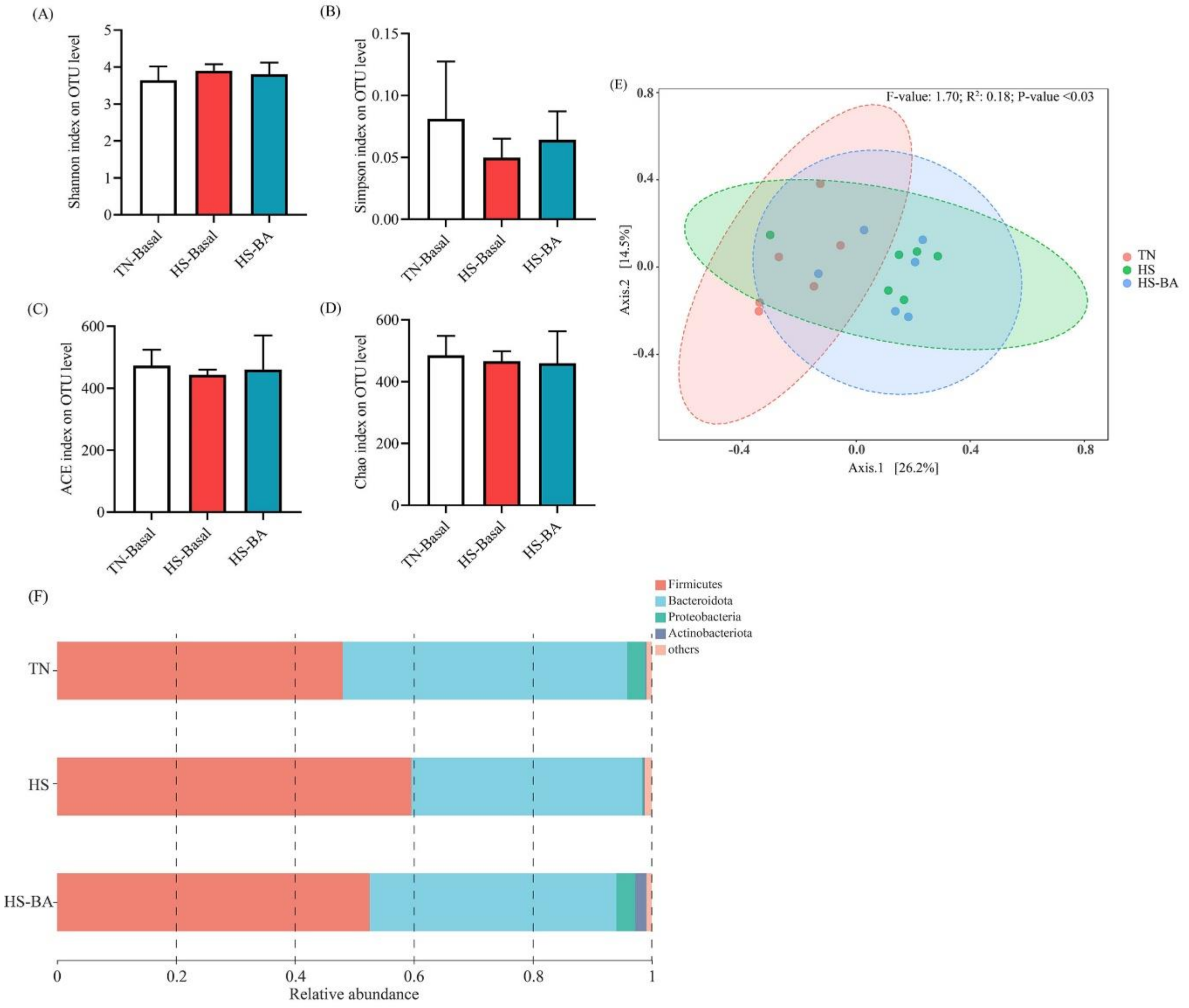

Figure S1 Heat stress alters the cecal microbiome. Alpha diversity using Shannon (A), Simpson (B), ACE (C), and Chao1. Beta diversity using PCoA plots based on Bray-Curtis dissimilarity matrices (E). (F) average phylum distribution of gut microbiomes in the TN, HS and HS-BA groups.TN, Thermoneutral; HS, Heat stress; BA, Bile acid supplements.

Table S1 The body weight of broiler chickens was evaluated at day 16 and day 21.

	Groups			*P*-value
	TN	HS	HS-BA	
16-d BW, g	469.88±46.34	469.76±46.32	469.76±46.52	1.0000
21-d BW, g	697.40±62.91	706.90±66.28	698.56±69.42	0.9640

All data are expressed as mean ± SD (n = 6 per group). In the same row, $P>0.05$ indicates no significant difference of three groups.

Table S2. Ingredient composition of the experimental diets (As-fed basis).

Items	0 ~ 21d	21 ~ 42d	21 ~ 42d
	Basal diet	Basal diet	BA diet
Ingredients (%)			
Corn	58.38	63.26	63.26
Soybean meal	27.50	22.00	22.00
Corn gluten meal	5.00	5.00	5.00
Soybean oil	4.00	4.00	4.00
$CaHPO_4$	1.80	1.80	1.80
NaCl	0.40	0.30	0.30
Limestone	1.30	1.30	1.30
Choline chloride	0.10	0.10	0.10
L-Lysine-HCl	0.22	0.18	0.18
DL- Methionine	0.20	0.16	0.16
L- Threonine	0.10	0.10	0.10
Premix[1]	1.00	1.00	1.00
Diatomite		0.8	0.8
Bile Acid Compound	-	-	0.02

[1] The nutrients provided per kilogram of premix as follows. Vitamin A, 5000 IU; Vitamin D, 3000 IU; Vitamin E, 75 mg; Vitamin K_3, 18.8 mg; Vitamin B_1, 9.8 mg; Vitamin B_2, 28.8 mg; Vitamin B_6, 19.6 mg; Vitamin B_{12} , 0.1 mg; Calcium pantothenate, 58.8 mg; Niacin, 196.0 mg; Folic acid, 4.9 mg; Biotin, 2.5 mg; Cu (as copper sulfate), 4.0 mg; Fe (as ferrous sulfate), 40.0 mg; Zn (as zinc sulfate), 37.6 mg; Mn (as manganese sulfate), 50.0 mg; Se (as sodium selenite), 0.2 mg; I (as potassium iodide), 0.2 mg.

Table S3. Primers of the tight junction proteins, inflammatory cytokines, and BA-related genes in ileum.

Gene[1]	GenBank ID	Primer sequence
FXR	NM_204113.2	F:5'-CAGAAAGAATGCAGCGGCTC-3'<br>R:5'-CAAACTGCCCCATTTTGCGA-3'
ASBT	NM_001319027.1	F:5'-CTCCCCTACGACAGCATTGG-3'<br>R:5'-GTGTGATAACCCACGAGCCT-3'
GLP-1	NM_205260.5	F:5'-TGGAAGGTCAAGCTGCCAAA-3'<br>R:5'-GGAAAACGTGAAGTACAGTAGGA-3'
AMPKα1	NM_001039603.1	F:5'-CGGAGATAAACAGAAGCACGAG-3'<br>R:5'-CGATTCAGGATCTTCACTGCAAC-3'
Keap-1	MN416132.1	F:5'-GTGGAGAGGTATGAGCCGGA-3'<br>R:5'-CTCGGTGCTGTTGAGCTGAT-3'
Nrf-2	XM_025152148.1	F:5'-GGCCACCCTAAAGCTCCATT-3'<br>R:5'-GGCTTCACTGAACTGCTCCT-3'
*i*NOS	NM_204961.1	F:5'-TCCTGAGTTCTGTGCCTTTG-3'<br>R:5'-GTTCATCTCCTTCACCCACTG-3'
TNFα	XM_040647309.1	F:5'-GAGCGTTGACTTGGCTGTC-3'<br>R:5'-AAGCAACAACCAGCTATGCAC-3'
β-actin	NM_205518.1	F:5'-TGTTACCAACACCCACACCC-3'<br>R:5'-TCCTGAGTCAAGCGCCAAAA-3'

[1] AMPKα1: adenosine 5'-monophosphate (AMP)-activated protein kinase-α1, ASBT: the apical ileal sodium-dependent bile acid cotransporter, FXR: farnesoid x receptor, GLP-1: glucagon-like peptide 1, iNOS: inducible NO synthase, Keap-1: kelch-like ECH-associated protein 1, Nrf2: nuclear factor-like 2, ZO-1: zonula occludens-1, TNFα: tumor necrosis factor-α.
